# Severe magnitude of dental and skeletal fluorosis and its impact on society and environment in a part of Manbhum-Singhbhum Plateau, India

**DOI:** 10.1186/s12889-024-19446-1

**Published:** 2024-07-23

**Authors:** Arijit Ghosh, Soumyajit Patra, Sumana Bhattacharjee, Biswajit Bera

**Affiliations:** 1 Department of Geography, Advanced Geomorphology and Hydrology Laboratory, Sidho-Kanho-Birsha University, Purulia, West Bengal India; 2 Department of Sociology, Sociology of Care Laboratory, Sidho-Kanho-Birsha University, Purulia, West Bengal India; 3 Department of Geography, Geomorphology and Environmental Geography Laboratory, Jogesh Chandra Chaudhuri College, Kolkata, West Bengal India

**Keywords:** Dental and skeletal fluorosis, Community Fluorosis Index (CFI), Dean’s Index, Manbhum-Singhbhum Plateau, Social consequence

## Abstract

**Background:**

Numerous approaches have been adopted to evaluate limited freshwater resources and the associated health hazards due to excessive amounts of fluoride in drinking water. The study aims to assess the degree and severity of dental and skeletal fluorosis and examine the broader effects of fluorosis on human health and society in the Manbhum-Singhbhum Plateau region, India.

**Methods:**

The Community Fluorosis Index (CFI) and Dean’s Index have been used to measure the magnitude and severity of dental and skeletal fluorosis. Questionnaire surveys, Focus Group Discussions (FGDs), and appropriate statistical methods have been applied to identify the social impacts. Risk-prone zones have been identified through overlay analysis using geoinformatics.

**Results:**

About 54.60% of people in 67 villages of this part of the Manbhum-Singhbhum Plateau are affected in varying degrees of fluorosis ranging from very mild to mild, moderate, and severe dental fluorosis. Among these 67 villages, Janra (Manbazar I) and Hijla (Barabazar) have the most severely affected people. School dropout (*n* = 426), social isolation (*n* = 149), remarriage (*n* = 21), and physically disabled (*n* = 75) have also been reported. The study shows that about 414.29 km^2^ of the Manbhum-Singhbhum Plateau comes under the high-risk-prone category.

**Conclusions:**

The societal and environmental awareness of the fluorosis-affected individuals is almost absent in this region. Economic hardships, lack of education, inadequate health care facilities, water scarcity, and lack of awareness increase the magnitude of health hazards and societal vulnerability of the people in this region, who are largely dependent on natural resources.

**Supplementary Information:**

The online version contains supplementary material available at 10.1186/s12889-024-19446-1.

## Introduction

Potable natural groundwater resources are vital for the advancement and progression of human society [1]. These limited freshwater resources play a noteworthy role in the maintenance of ecosystem services and act as an open system that is associated with other aquatic and terrestrial ecosystems [2]. Almost 2.5 billion people of the world are dependent on groundwater to fulfill their daily needs. Therefore, the supply of good groundwater in a proper way is a precondition of sustainable livelihood systems [3, 4].The physiological and biological activities of human beings are extremely reliant on these freshwater resources; but climate change and excess modifications of land use and land cover trigger the challenges to access good quality freshwater from different sources worldwide [5]. In recent decades, underground storage of freshwater has been reduced in many semi-arid highly populated areas of the world. It exaggerates the effluences in the freshwater [6].For example, the amount of fluoride in drinking water sources in semi-arid environments is a major concern that is gradually increasing due to the decline of groundwater storage [7–11].Climate change, geogenic sources and anthropogenic activities viz. fluoride-rich rock-water interaction, excess use of fertilizers, dumping of industrial waste, domestic sewage and excessive pumping of groundwater lead to the fluoride content in groundwater aquifer [11–14].

Globally, 200 million people in 29 nations are facing severe fluoride-related health hazards, while in India, 30% of people are affected by these hazards. Recent estimates show that in India, almost 60 million people in 21 states are in front of this hazard [15, 16].These fluoride-related health issues crop up due to high concentrations of fluoride ions in groundwater. Recently, this toxicological environmental risk has been amplified as a result of warm climatic conditions [17, 18]. Fluoride concentration in drinking water crosses the optimum level (< 1.5 mg/L, WHO) in different environmental regions. This high level of fluoride accelerates several detrimental impacts on the human body (Kisku and Sahu, 2020) which in turn are manifested in different social consequences [1].

The health impact due to fluoride is generally called as ‘fluorosis’ which arises from excess fluoride intake through drinking water. It affects the teeth (known as dental fluorosis), bones (known as skeletal fluorosis), and soft tissues (known as non-skeletal fluorosis) of the human body [19, 20]. Primary symptoms start from dental fluorosis; and it is associated with pitting, perforation and chipping of the teeth [21]. Moreover, crippling, deformities and osteoporosis of bones are associated with skeletal fluorosis [21, 22]. Even sometimes it leads to paralysis as an ultimate consequence. Neurological disorders including muscular manifestations and gastro-intestinal problems also happen due to excess intake of fluoride ions through drinking water [23–25]. Besides these devastating health complications, school dropout, stigma and social rejections, isolation, job loss, physical disability, divorce, remarriage, and dowry system are attached with fluorosis hazard as societal consequences [11]. Sociologists [26] try to estimate the economic costs (for example, loss of job and productivity) and social costs (for example, social isolation) of health problems alongside its biological cost (for example, neurological disorders). The problem is that, more often than not, people cannot identify the problem as a ‘*problem*’ at all. This is particularly true of the tribal people, who have a particular mind set. Bakhle (1980), in this regard, has distinguished between ‘problems of the tribals’ (external problems like fluoride contamination) and ‘problems with the tribals’ (internal or psychological problems like their lack of awareness) [27]. A complex interplay of these two aggravates the health problems and affects the society at large. ‘Health’, in this study, has been conceptualized following the definition given by the constitution of World Health Organization (WHO): ‘Health is a state of complete physical, mental and social well-being and not merely the absence of disease or infirmity’ [28].

Several hydrological investigations related to the distribution of fluoride concentration and identification of sources have been conducted by the researchers but specific studies on health and social consequences are still inadequate. Field-based, intensive and detailed study is essential in this context to fulfill the research gap. In this connection, geogenic sources of fluoride and its direct and indirect impacts on human health and society have been studied in this work. Few researchers have conducted studies in several high fluoride-affected states of India including Assam, Andhra Pradesh, Bihar, Chhattisgarh, Gujarat, Haryana, Karnataka, Kerala, Maharashtra, Madhya Pradesh, Odisha, Punjab, Rajasthan, Tamil Nadu, Uttar Pradesh and West Bengal. These states are severely affected by fluoride and physical health hazards and concomitant societal problems [21]. Additionally, dental fluorosis is the first indication of the difficulties of fluoride-affected people. Identification of the stages of dental fluorosis is important to manage the severity of fluorosis in a fluoride-affected region. In this study, the dental conditions of the villagers have been observed during the fieldwork in 67 villages where we found a high amount of fluoride in groundwater. Dean’s Index has been considered to assess the stages of severity among the affected people of different villages of Purulia district [28, 29].In addition, symptoms of skeletal fluorosis are calcification of ligaments, bone sickness, or overall variations in bone structure. These health issues arise due to high fluoride intake through drinking water or other sources [30]. Excess fluoride is accumulated in the joints of the knees, bones of the shoulder, and neck including the pelvic area, and it triggers difficulties in regular activities, particularly in walking [1]. As per the report of the World Health Organization (WHO, 2002), this accumulation of fluoride varies with age and bone types [31]. Consuming water with high fluoride (above 5 mg/L) is the responsible factor for crippling skeletal fluorosis. This skeletal fluorosis is marked by kyphosis, scoliosis, flexion deformity, paraplegia, and quadriplegia. Kyphosis is the unusual convex shape in the mid-sections of the spine whereas scoliosis is the lateral curve in the spine [1]. Flexion deformity means curvature in the knee and inability to straighten it fully. Paraplegia is paralysis due to dysfunctions of lower body muscles whereas Quadriplegia is the paralysis of four limbs of the human body [8].

In the state of West Bengal, about 43 community blocks in seven districts including extended sections of the Manbhum-Singhbhum Plateau (Purulia, Bankura and Birbhum) are affected by fluoride contamination in drinking water sources. Maximum number of people in this plateau fringe face fluoride-related health difficulties due to the consumption of high fluoride-contaminated water for their survival. Several researchers have conducted studies on fluoride concentration, distribution and sources in different affected areas including the Chota Nagpur plateau [13, 32, 33]. Most of the studies are confined to hydrological investigations and there is a gap in terms of assessment of the impacts on health and society. Therefore, this field-based door-to-door survey has been conducted to assess the magnitude of its effects on both human health (dental and skeletal fluorosis) and society. Thus, the principal objectives of this study are i) to assess the magnitude of dental and skeletal fluorosis among the 20 blocks of Purulia district, an extended section of the Manbhum-Singhbhum Plateau, and ii) to inspect the effects of fluoride on human health and society and to categorize the risk-prone zones in this tribal-dominated district of West Bengal. The novelty of this work is the examination of the ways in which various societal problems are unfolded and linked with groundwater fluoride contamination. This study also points out that many poor tribal families are being excluded from the so-called ‘mainstream’ society because of severe economic distress and livelihood vulnerabilities. These research findings will definitely assist the policy makers and administrators who are working for tribal development and sustainable environmental management.

## Methods and materials

### Fundamental information of the study area

**Fig. 1:**
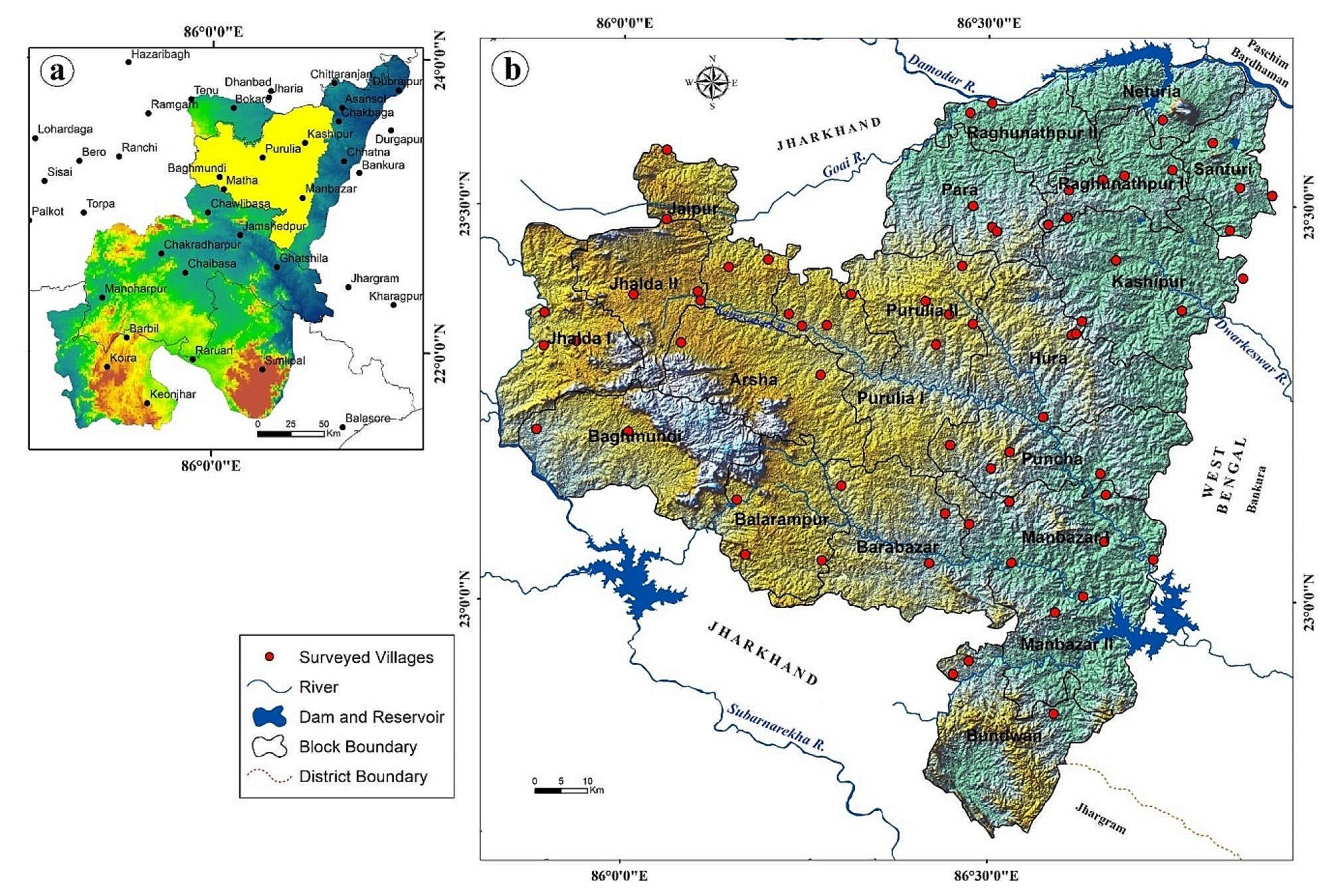
**a**) Purulia district in Manbhum-Singhbhum Plateau region **b**) Surveyed villages under different blocks of Purulia district with hydrological aspects

Manbhum-Singhbhum Plateau is situated in the eastern section of the Chota Nagpur Plateau [34]. This is in the part of Singhbhum Protocontinent in the Indian Peninsular Shield. Therefore, the principal rocks are granite, gneiss and pegmatite [35]. Geographically, the study district lies in between north latitude of 22°42’  35´´ to 23°42’  00´´ and east longitude of 85°49’ 25´´ to 86°54’ 37´´. Purulia district covers 20 Community Development Blocks and it is bounded by Paschim Bardhaman, Bankura, and Jhargram districts of West Bengal from northeast, east, and southeast, whereas, the northwestern, western and southwestern sides are encompassed by the Jharkhand state of India (Fig. 1). The entire region is one of the vulnerable zones in Singhbhum-Manbhum Plateau in terms of fluoride-contaminated groundwater [10]. In this region, many fluoride-bearing minerals like fluorite, biotite and apatite are present [36]. These geogenic sources are responsible for high fluoride content in groundwater because of their locations in a semi-arid plateau fringe with fluoride-contained minerals. Physical as well as chemical weathering processes are more active in this climatic region. Here, the average annual temperature varies from 23 °C to 39 °C whereas the average rainfall is 1200 mm approximately. The scarcity of fresh water is a major concern in this area. However, rivers like Subarnarekha, Kangsabati, Kumari and Damodar play an important role in water crisis management in this dry environment, particularly during the summer season [37]. It should be noted that most of the villagers here are tribals and are largely dependent on agricultural activities through irrigation.

### Clinical survey and community fluorosis index (CFI) for dental fluorosis

The entire study is ethically reviewed and approved by the Institutional Ethical Committee (memo no. is R/IEC/280/SKBU/24) and ‘Informed consent to participate’ was obtained from all studied participants and individuals who are younger than the age of 16 and obtained also from their parents. After appropriate cleaning, dental loupes were used carefully to examine the teeth of the surveyed people (*n* = 1017) in daylight. These proper procedures have been taken during the examination to get accurate results because it may be mistaken as stains developed on their teeth due to chewing tobacco and smoking. Dean classified the dental fluorosis studies as normal, questionable, very mild, mild, moderate, and severe. Numerical weightage values have been considered as per Dean’s Index viz. 0, 0.5, 1, 2, 3 and 4 separately. Detailed criteria of this classification have been displayed in Table 1. The magnitude and intensity of dental fluorosis have been identified and classified based on the above criteria. The Community Fluorosis Index (CFI) has been estimated through the mean values of scores using equation no. 1.

**Table 1:** Criteria for Dean’s classification system for Dental Fluorosis [38]

Classification and Score	Criteria
Normal (0)	Generally, enamel symbolizes translucent semivitri structure. The surface is smooth and glossy with white colour.
Questionable (0.5)	The enamel shows minor differences from the translucency of usual enamel, ranging from a few white specks to occasional white Empots. This classification is used in cases where an obvious diagnosis of the mildest kind of fluorosis is not necessary and “normal” is not acceptable
Very Mild (1)	Small, opaque, paper-white regions are spread arbitrarily around the teeth but do not encompass more than 25% of the surface. This categorization frequently includes teeth with no more than 1–2 mm of white opacity at the tip of the top of the cusps of the bicuspids or second molars.
Mild (2)	The white opaque patches in the enamel of the teeth are more widespread but do not encompass more than 50% of the tooth.
Moderate (3)	All teeth enamel surfaces are exaggerated and attrition-prone surfaces show worn. The brown stain is commonly a disfiguring feature.
Severe (4)	All surface of enamel is affected and hypoplasia is noticeable. The normal form of the tooth may be affected. The main diagnostic sign of this classification is confluent pitting. Brown stains are prevalent and teeth often have a corroded appearance.

1 $$CFI = \sum {\frac{{Number\,of\,people \times \,Dean's\,numerical\,weight}}{{Total\,number\,of\,people}}} $$

Here, ‘number of people’ signifies the number of affected people and ‘total number of people’ means total number of people who have been considered to assess the severity of dental fluorosis. CFI values of 0-0.4, 0.4–0.6, 0.6-1, 1–2,2–3 and 3-4 indicate a ‘negative’, ‘borderline’, ‘slight’, ‘medium’, ‘marked’, and ‘very’ marked public health concern respectively. It should be mentioned here that when the value crosses 0.6, fluorosis becomes a public health issue in those regions.

### Clinical survey and analysis of skeletal fluorosis

In this study, evaluation of the severity and stages of skeletal fluorosis has been done through three basic steps such as touching the toenail without twisting the knees, touching the torso with the jaw, and after stretching the arms laterally bending them towards the head from the back. However, these exercises are not performed in the case of surveyed people who have already had stiffness with pain in the backbone, joints, and hip [39, 40]. Finally, the severity and magnitude of skeletal fluorosis have been measured using the grade methods which were proposed by Teotia et al. (1986). General bone and joint pains are considered as mild skeletal fluorosis, stiffness, and rigidity with spine and joint movement difficulties are considered as moderate skeletal fluorosis. In addition, these two difficulties include deformities of limbs and spine, knock knees, and crippling and bedridden state are considered as severe skeletal fluorosis [41].

### Sampling and Focus Group Discussion to assess the societal impact assessment

To assess the direct and indirect impacts of fluoride on society, a total of 67 villages have been selected (fluoride greater than 1.5 mg/L) with 478 households surveyed in this fluoride-contaminated district of West Bengal . Out of the total surveyed population (5365), 3066 are males (57.15%) and 2299 (42.85%) are females. In this study, stratified random, systematic, and quota sampling methods have been applied to collect the data from the respondents of the selected villages, and an appropriately structured questionnaire has been designed. An open-ended questionnaire has been developed to know some significant information viz. demographic characteristics, water quality and magnitude of fluorosis and food habits, medical facility, and management. Statistical Pearson correlation and analysis have been done using Python script in Jupyter Notebook to assess the magnitude of the social impact of high fluoride in groundwater. In addition, Focus Group Discussions (FGDs) have been used to collect semi-structured data through the discussions on the concerning issues [42]. It was more beneficial to collect data on the difficulties faced by the people of fluoride-affected areas of this extended section of the Manbhum-Singbhum Plateau fringe. However, this method is extremely popular as a faster way to acquire information from the target people [43]. However, this method has been applied frequently in this study to collect qualitative data for health and social impact assessment or understanding of societal issues [44]. Correlations have been assessed to know the relationship among the fluoride concentrations in different surveyed villages of the study region.

### Identification of risk zones through weightage overlay analysis

In this study, risk zones have been identified based on some important parameters such as village-level fluoride concentrations, percentage of dental fluorosis cases, percentage of skeletal fluorosis cases, safe drinking water facilities, density of population, and distance from the hospital. Data on safe drinking water facilities have been collected from the Public Health Engineering Department, Govt. of West Bengal (https://www.wbphed.gov.in). In addition, names of rural and Sadar hospitals have been collected from the Department of Health and Family Welfare, West Bengal (https://www.wbhealth.gov.in) for the calculation of the distance of contaminated villages from the hospitals. All thematic layers and weightage overlay analyses have been performed using ArcGIS software. This method is more effective in terms of multi-criteria decision approaches [45]. In this raster overlay method, each cell has the same size (30 × 30 m) and is referenced in the same projections (Projected Coordinate System). Weight values (1 to 5) have been assigned for each factor thematic layer based on their importance to produce an output raster of risk zonation map.

## Result

### Effect of Community Fluorosis Index (CFI) and dental fluorosis

**Fig. 2:**
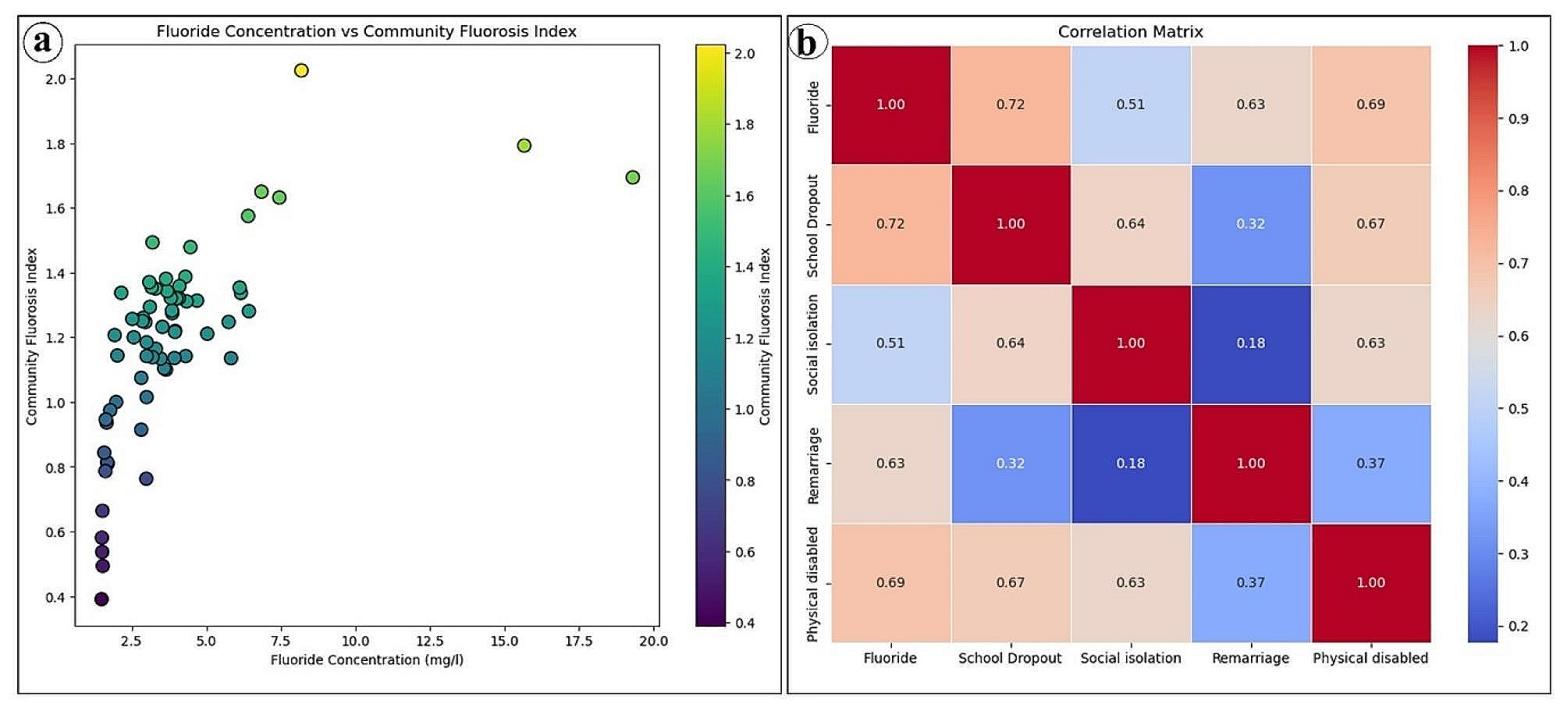
**a**) Scatter plot among the fluoride concentration and community fluorosis **b**) Correlation matrix (Pearson’s correlation method) among the fluoride concentration and social effects over the different parts of the Purulia District

**Fig. 3:**
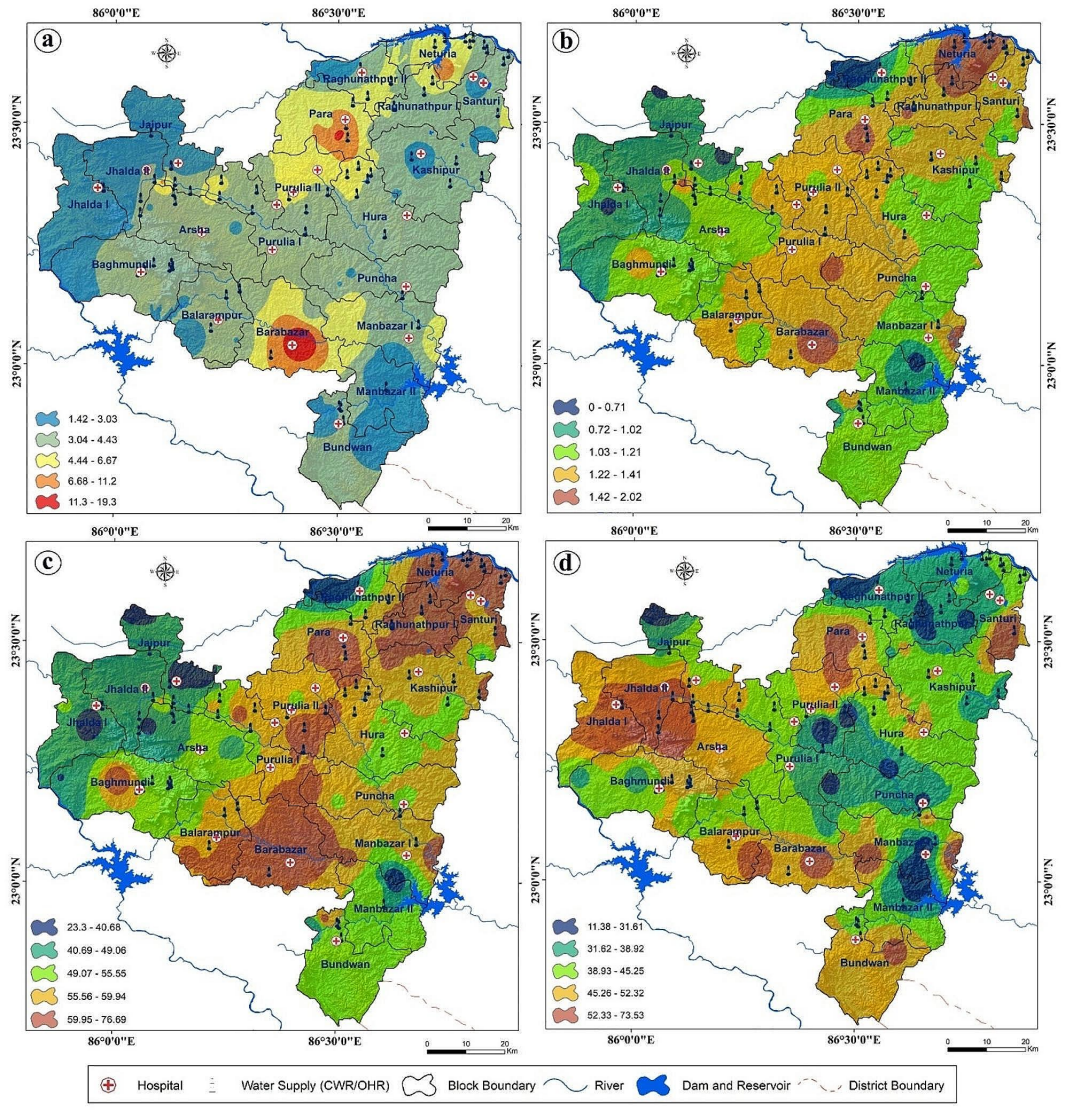
**a**) Fluoride concentration zones **b**) Community fluorosis index **c**) Percentage of dental fluorosis cases (mild to severe) and **d**) Percentage of skeletal fluorosis cases (mild to severe) concerning collected samples over this part of Manbhum-Singhbhum Plateau region

Dental fluorosis survey data of selected highly contaminated villages reveal the extensive prevalence of fluorosis among the villagers in this section of the plateau fringe (Fig. 2). Both males and females including children are affected by dental fluorosis. The Community Fluorosis Index based on total affected cases indicates that Kachbel village (2.02) has the highest fluorosis-affected area. However, the result expresses that out of the total surveyed villages, Jabarra village (77.53%) faces the highest percentage of dental fluorosis cases. On the other hand, Ichhar village (0.39) has the lowest Community Fluorosis Index which denotes the lowest prevalence and severity of dental fluorosis. In addition, in this village, 23.08% of the surveyed people are affected by different stages of dental fluorosis. In this study region, the average Community Fluorosis Index value is 1.17 which indicates that the prevalence and severity of dental fluorosis are highly concerning issues. About 54.60% of people in 67 villages of this part of the Manbhum-Singhbhum Plateau are impacted by very mild, mild, moderate, or severe dental fluorosis. Following villages of this part viz. Kachbel, Jabarra, Hijla, Marbedya, Nagra, Khayerbani, Penchara, Rangametya, Chinpina have CFI values of 2.02, 1.79, 1.69, 1.65, 1.63, 1.57, 1.49, 1.48 and 1.39 respectively. But the five low affected villages are Ichhar, Santaldih, Chalka, Agharpurand Gokulnagar with CFI values of 0.39, 0.46, 0.49, 0.54, and 0.58 respectively. The percentage of dental fluorosis cases is alarming in Jabarra, Kachbel and Marbedya villages where the percentage crosses 70%. In Hijla village (Balarampur block) 68.37% of people have very mild, mild, moderate or severe dental fluorosis. Village-wise Community Fluorosis Index value and percentage of dental fluorosis have been shown in Table 2. The Community Fluorosis Index map reveals that northwestern, south-western, and some extreme northern portions of this part of the Manbhum-Singbhum Plateau face less dental fluorosis than the other portions of this study area (Fig. 3b). But in terms of block-wise data, Para, Barabazar, Raghunathpur I, Purulia I, and Purulia II and Nituria come to the list of high fluoride contamination in drinking water. Significantly, surrounding areas of Ajodhya hill range experience nominal dental fluoride cases (Fig. 3c).

**Table 2:** Community Fluorosis Index (CFI) and percentage of Dental fluorosis (DF) among the fluoride-contaminated villages of Purulia district

Name of the village	Fluoride Concentration (mg/L)	CFI	% DF	Name of the village	Fluoride Concentration (mg/L)	CFI	% DF
Agharpur	1.51	0.54	29.63	Janra	5.04	1.21	61.45
Asanbani	3.47	1.13	59.70	Jaipur	1.62	0.79	35.29
Bagatbari	3.20	1.14	55.26	Jhanpara	6.16	1.34	55.43
Bamu	3.30	1.35	59.77	Jibanpur	4.31	1.14	54.32
Banahata	1.63	0.95	43.08	Jorabel	3.93	1.14	65.22
Belma	4.69	1.31	55.42	Kachbel	8.19	2.02	73.49
Benrya	1.98	1.00	48.21	Karkara	1.52	0.66	37.50
Bhangra	4.08	1.32	59.26	Kenda	2.88	1.26	56.00
Bhul	4.10	1.36	64.86	Keshargaria	5.75	1.25	55.91
Birsingdi	3.53	1.23	59.52	Ketuabera	3.59	1.10	49.35
Brahmandi	3.00	1.02	46.97	Khayerbani	6.40	1.57	65.52
Chalka	1.53	0.49	30.00	Kusumjuria	2.86	1.25	55.41
Chandra	1.68	0.81	41.43	Lapara	2.15	1.34	59.04
Chaupad	2.57	1.20	50.67	Mahara	3.95	1.22	65.56
Chharra	5.83	1.14	52.94	Malti	2.52	1.26	60.81
Chinpina	4.30	1.39	58.33	Marbedya	6.85	1.65	73.20
Chirudi	2.02	1.14	55.00	Nagra	7.45	1.63	58.95
Deorang	2.96	1.25	54.02	Nishchintapur	3.65	1.38	54.55
Durgu	1.58	0.84	45.31	Patamputra	6.43	1.28	59.18
Fatehpur	3.00	1.14	61.73	Pathraghata	4.34	1.31	62.20
Garga	3.00	1.18	52.31	Penchara	3.20	1.49	61.64
Garurbasa	3.96	1.22	58.54	Phusura	3.11	1.29	62.89
Garurbasa	2.82	0.91	48.78	Puncha	3.65	1.10	53.75
Gobindapur	3.81	1.32	64.29	Ramchandrapur	1.93	1.21	58.67
Gokulnagar	1.50	0.58	32.35	Ramkanali	3.69	1.34	64.04
Gordih	3.17	1.35	64.56	Rangametya	4.47	1.48	59.14
Guridi	1.66	0.94	44.83	Salberya	3.31	1.16	57.14
Gurur	3.09	1.37	61.73	Saldabar	1.70	0.81	40.51
Hesla	2.82	1.07	44.78	Santaldih	1.41	0.46	30.59
Hijla	19.30	1.69	68.37	Sharbarya	6.12	1.35	63.64
Hulka	3.86	1.27	58.54	Tamakhun	1.78	0.97	51.90
Hutmura	3.85	1.28	61.96	Tarang	4.00	1.32	65.33
Ichhar	1.49	0.39	23.08	Upargugui	2.99	0.76	38.16
Jabarra	15.66	1.79	77.53				

### Scenario of skeletal fluorosis variation and magnitude

The survey results show that the numbers of skeletal fluorosis viz. mild, moderate and severely affected people are 1425,792, and 87 respectively. However, 3061 people have no such symptoms among the surveyed population (total of 5365). Now, block-wise variations at the village level data are shown. Fluorosis-related health hazards are lowest in Hutmura village (Purulia II) where 74 persons have no fluorosis sign among the 92 surveyed people. However, the highest number of mild skeletal fluorosis cases are found in Khayerbani village (Manbazar I, *n* = 49 ) whereas the lowest is in Chalka village (Manbazar II, *n* = 8). In terms of moderately affected people, the highest number of people (27) come in Sharbarya village (Para) of Purulia district. On the other hand, Karkara village (Jaipur) has no moderate skeletal fluorosis cases. In addition, Janra (Manbazar I) and Hijla (Barabazar) villages have the highest number of severely affected people among the 67 surveyed villages (Table 3). Six people in each village are facing difficulties due to chronic skeletal fluorosis, among them two villagers have migrated to another village with their family members (Table 3). However, the result shows that 28 surveyed villages have no severe fluorosis cases. The percentages of the total affected people (out of the total surveyed people of that particular village) are 9.76% in Garurbasa (Hura) village followed by Janra (7.23%) and Hijla (6.12%) in Purulia. The maximum moderate cases are found in Benrya (Santuri) village (39.29%) followed by Fatehpur (Balarampur) (32.31%) and Banahata (Jhalda II) (32.10%) whereas the maximum umber of mild skeletal fluorosis are found in Belma (Purulia II) (56.32%) followed by Salberya (Santuri) (52.86%) andKhayerbani (50.60%). Karkara village (88.75%), Gobindapur (Raghunathpur I) (85.71%) and Chalka (82.81%) have been found having more non-fluorosis affected people than the other surveyed villages in this extended section of the Manbhum-Singbhum Plateau.

**Table 3:** Village-wise detailed cases and grade of skeletal fluorosis in the part of the Manbhum-Singhbhum Plateau region

Name of the Village	Fluoride concentration (mg/L)	Normal %	Mild%	Moderate%	Severe%	Name of the Village	Fluoride concentration (mg/L)	Normal %	Mild %	Moderate%	Severe%
Agharpur	1.5	55.6	19.8	19.8	4.9	Janra	5.0	33.7	37.3	21.7	7.2
Asanbani	3.5	46.3	35.8	13.4	4.5	Jaipur	1.6	58.8	20.6	20.6	0.0
Bagatbari	3.2	64.5	14.5	18.4	2.6	Jhanpara	6.2	47.8	45.7	6.5	0.0
Bamu	3.3	60.9	29.9	9.2	0.0	Jibanpur	4.3	64.2	19.8	13.6	2.5
Banahata	1.6	38.5	29.2	32.3	0.0	Jorabel	3.9	50.0	33.7	15.2	1.1
Belma	4.7	37.3	50.6	8.4	3.6	Kachbel	8.2	60.2	30.1	9.6	0.0
Benrya	2.0	35.7	21.4	39.3	3.6	Karkara	1.5	82.8	17.2	0.0	0.0
Bhangra	4.1	45.7	29.6	22.2	2.5	Kenda	2.9	64.0	28.0	8.0	0.0
Bhul	4.1	60.8	14.9	23.0	1.4	Keshargaria	5.8	52.7	18.3	24.7	4.3
Birsingdi	3.5	73.8	21.4	3.6	1.2	Ketuabera	3.6	55.8	35.1	9.1	0.0
Brahmandi	3.0	45.5	36.4	16.7	1.5	Khayerbani	6.4	33.3	56.3	8.0	2.3
Chalka	1.5	88.8	10.0	1.3	0.0	Kusumjuria	2.9	58.1	36.5	5.4	0.0
Chandra	1.7	48.6	24.3	25.7	1.4	Lapara	2.2	54.2	28.9	15.7	1.2
Chaupad	2.6	54.7	25.3	20.0	0.0	Mahara	4.0	78.9	14.4	6.7	0.0
Chharra	5.8	49.4	30.6	18.8	1.2	Malti	2.5	52.7	23.0	23.0	1.4
Chinpina	4.3	64.3	17.9	11.9	6.0	Marbedya	6.9	55.7	32.0	12.4	0.0
Chirudi	2.0	45.0	28.8	26.3	0.0	Nagra	7.5	49.5	25.3	20.0	5.3
Deorang	3.0	62.1	29.9	8.0	0.0	Nishchintapur	3.7	71.6	21.6	5.7	1.1
Durgu	1.6	46.9	23.4	28.1	1.6	Patamputra	6.4	46.9	27.6	21.4	4.1
Fatehpur	3.0	42.0	23.5	32.1	2.5	Pathraghata	4.3	53.7	29.3	17.1	0.0
Garga	3.0	58.5	20.0	21.5	0.0	Penchara	3.2	71.2	19.2	8.2	1.4
Garurbasa	4.0	70.7	18.3	11.0	0.0	Phusura	3.1	71.1	25.8	3.1	0.0
Garurbasa	2.8	26.8	39.0	24.4	9.8	Puncha	3.7	75.0	16.3	8.8	0.0
Gobindapur	3.8	85.7	13.1	1.2	0.0	Ramchandrapur	1.9	68.0	12.0	18.7	1.3
Gokulnagar	1.5	26.5	45.6	26.5	1.5	Ramkanali	3.7	73.0	20.2	6.7	0.0
Gordih	3.2	68.4	26.6	5.1	0.0	Rangametya	4.5	58.1	18.3	22.6	1.1
Guridi	1.7	63.2	24.1	11.5	1.1	Salberya	3.3	30.0	52.9	15.7	1.4
Gurur	3.1	60.5	17.3	21.0	1.2	Saldabar	1.7	63.3	16.5	17.7	2.5
Hesla	2.8	49.3	26.9	19.4	4.5	Santaldih	1.4	74.1	17.6	7.1	1.2
Hijla	19.3	38.8	38.8	16.3	6.1	Sharbarya	6.1	34.3	35.4	27.3	3.0
Hulka	3.9	61.0	29.3	8.5	1.2	Tamakhun	1.8	65.8	13.9	20.3	0.0
Hutmura	3.9	80.4	16.3	3.3	0.0	Tarang	4.0	65.3	28.0	6.7	0.0
Ichhar	1.5	75.6	21.8	2.6	0.0	Upargugui	3.0	35.5	40.8	23.7	0.0
Jabarra	15.7	52.8	30.3	12.4	4.5						

**Fig. 4:**
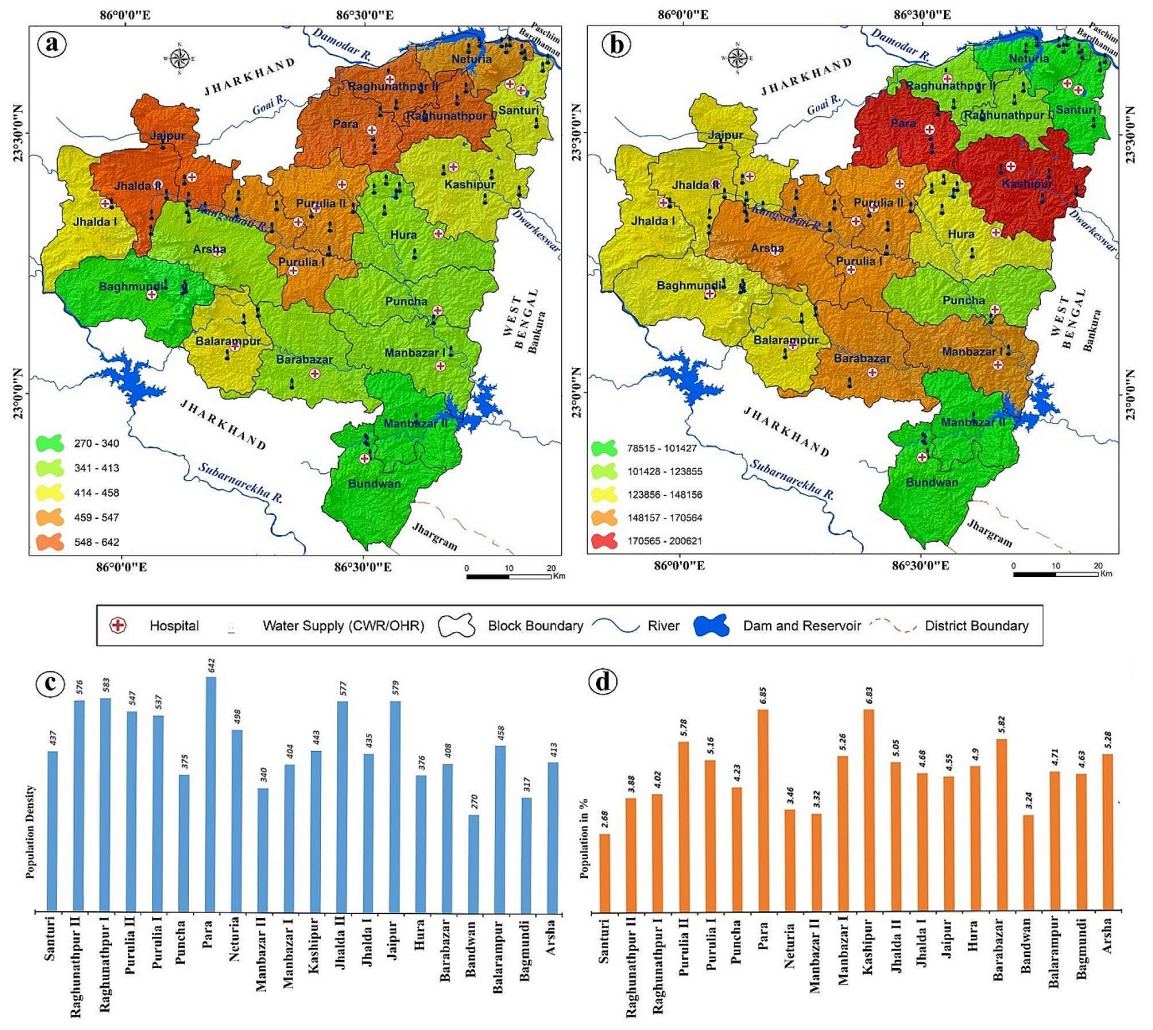
**a**) Population density map as per Census of India, 2011 and **b**) Population distribution map **c**) Graphical representation of population density variations and **d**) Number of population in different blocks of Purulia district in the extended section of Manbhum-Singbhum Plateau

### Signature of fluorosis in society

Fluorosis diminishes work efficiency (and reduces the productivity of the individual which indicates its economic cost) and influences the quality of life (and affects social participation and social relationships indicating its social cost) which directly or indirectly generates numerous complications (including mental health issues that indicate its psychological cost) in normal livelihood systems. After the assessment of societal impacts, the result expresses that school dropout (*n* = 426) is the foremost concern of fluorosis followed by social isolation (*n* = 149), remarriage (*n* = 21), and physical disability (*n* = 75) in this plateau fringe area (Table 4). It has been found that Nagra village of Arsha block (*n* = 22) has the maximum number of school dropouts followed by Kachbel of Naturia block (*n* = 19) and Hijla of Barabazar (*n* = 18) whereas Tamakhun of Manbazar II (*n* = 1), Chalka of Manbazar II (*n* = 1), Karkara of Jaipur (*n* = 1) and Santaldih of Raghunathpur II (*n* = 1) villages have lowest school dropout (Table 4). Nagra (Arsha) and Jibanpur (Kashipur) villages (*n* = 8) have the highest number of socially isolated people followed by Kachbel (Neturia), Patamputra (Purulia I), Pathraghata (Barabazar), Bhangra (Purulia II), and Chinpina (Raghunathpur I) (*n* = 7). A total of 41 among the surveyed villages have cases of physical disabilities in this consecutive manner viz. Hijla of Balarampur (6), Jabarra of Para (6), Nagra of Arsha (5), Janra of Manbazar I (5), Chinpina of Raghunathpur I (5) (Table 4). At the same time, cases of remarriage due to fluorosis are found only in 15 surveyed villages. The average school dropout rate is 6.30% among the 67 household observations.

**Table 4:** Social impact assessment result of different villages in this part of Manbhum-Singhbhum Plateau

Name of the Village	Fluoride concentration (mg/L)	No. of School Dropout	No. of Social isolation	No. of Remarriage	No. of Physical disability	Village	Fluoride (mg/L)	No. of School Dropout	No. of Social isolation	No. of Remarriage	No. of Physical Disability
Agharpur	1.5	2	0	0	1	Janra	5	10	2	0	4
Asanbani	3.5	3	3	1	2	Jaipur	1.6	6	0	0	0
Bagatbari	3.2	6	2	0	1	Jhanpara	6.2	9	1	0	0
Bamu	3.3	4	2	0	0	Jibanpur	4.3	7	8	0	1
Bachata	1.6	2	2	0	0	Jorabel	3.9	5	4	0	1
Belma	4.7	5	2	1	2	Kachbel	8.2	19	7	2	1
Benrya	2	5	1	0	1	Karkara	1.5	1	1	0	0
Bhangra	4.1	9	7	0	2	Kenda	2.9	3	1	0	0
Bhul	4.1	3	4	0	1	Keshargaria	5.8	5	4	1	3
Birsingdi	3.5	10	2	0	1	Ketuabera	3.6	7	2	0	0
Brahmandi	3	8	2	0	1	Khayerbani	6.4	12	4	0	2
Chalka	1.5	1	0	1	0	Kusumjuria	2.9	5	1	1	1
Chandra	1.7	4	2	1	1	Lapara	2.2	4	1	0	1
Chaupad	2.6	2	1	0	0	Mahara	4	3	1	0	0
Chharra	5.8	8	2	0	1	Malti	2.5	5	2	0	1
Chinpina	4.3	8	7	0	4	Marbedya	6.9	18	4	0	3
Chirudi	2	2	2	0	0	Nagra	7.5	22	8	0	5
Decorating	3	2	0	0	0	Nishchintapur	3.7	8	1	0	1
Durgu	1.6	3	1	0	1	Patamputra	6.4	14	7	1	3
Fatehpur	3	5	1	1	2	Pathraghata	4.3	9	7	0	1
Garga	3	6	1	0	2	Penchara	3.2	5	1	0	0
Garurbasa	4	6	1	0	0	Phusura	3.1	9	0	0	0
Garurbasa	2.8	8	2	0	2	Puncha	3.7	3	0	0	0
Gobindapur	3.8	3	1	0	0	Ramchandrapur	1.9	3	1	0	1
Gokulnagar	1.5	4	0	1	0	Ramkanali	3.7	6	1	1	0
Gordih	3.2	8	0	0	0	Rangametya	4.5	7	3	0	1
Guridi	1.7	5	1	0	1	Salberya	3.3	8	2	0	1
Gurur	3.1	4	2	0	1	Saldabar	1.7	2	2	0	1
Hesla	2.8	6	2	0	2	Santaldih	1.4	1	0	0	0
Hijla	19	18	6	2	6	Sharbarya	6.1	6	3	1	2
Hulka	3.9	7	2	0	1	Tamakhun	1.8	1	1	0	0
Hutmura	3.9	5	1	2	0	Tarang	4	8	1	0	0
Ichhar	1.5	3	0	0	0	Upargugui	3	6	3	0	1
Jabarra	16	14	3	4	4						

### Status of fluorosis risk zonation

**Fig. 5:**
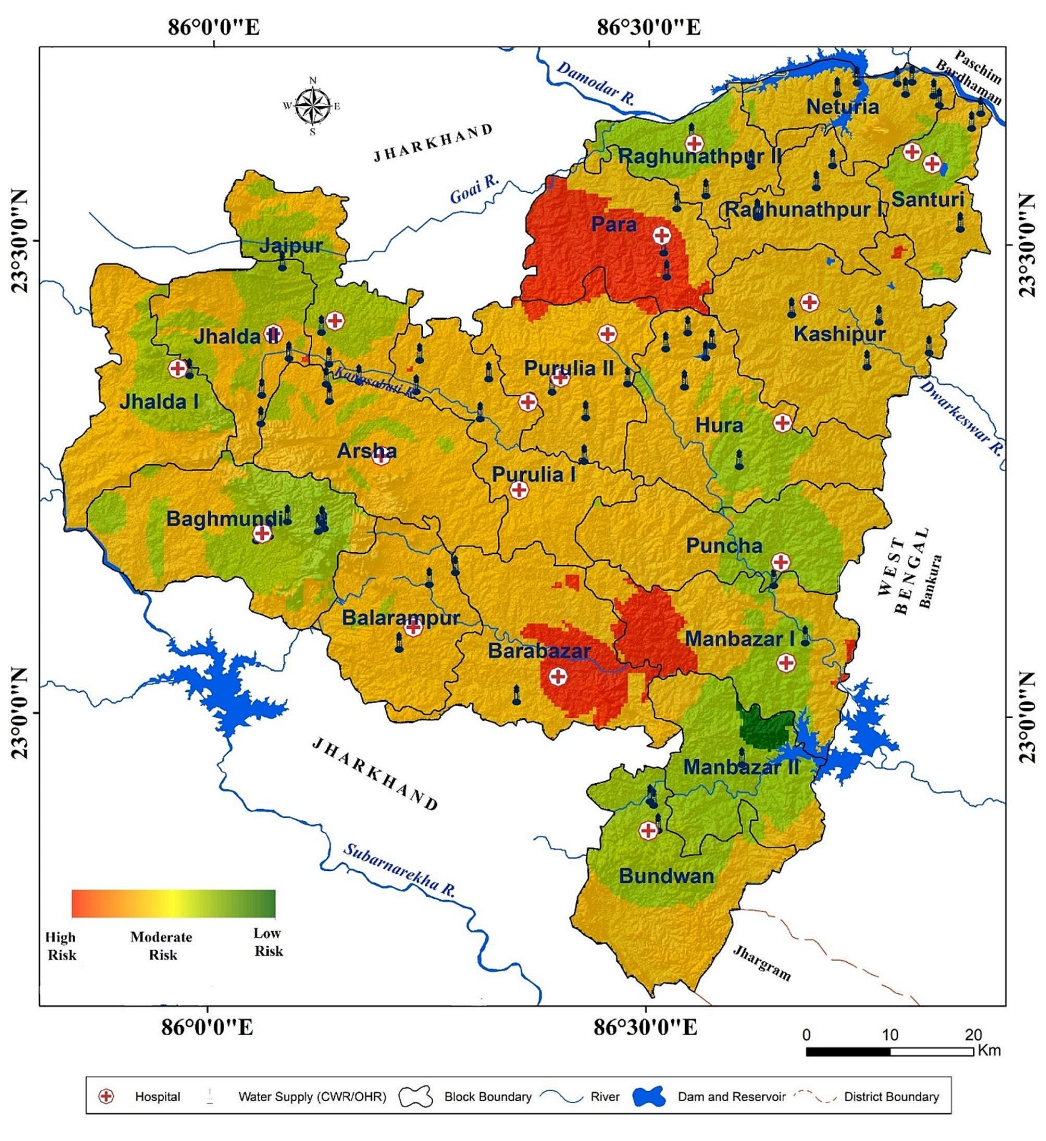
Fluoride risk zones (high to low) of Purulia district, a part of Manbhum-Singhbhum Plateau

Risk zone identification has been done using six important controllers of the endemic in this eastern section of the Manbhum-Singbhum Plateau. These six parameters are extremely connected to recognize the susceptibility due to fluoride exposure, prevalence and severity. Medical facilities, alternative drinking water sources, affected dental and skeletal fluorosis cases, and population density were used to demarcate the risk areas in this fluoride-affected hotspot region (Fig. 4). A total of 20 hospital data including Deben Mahato Hospital (Hatuara) and Purulia Sadar Hospital (Purulia) were used to assess the medical facilities among the affected people in contaminated villages. In addition, 66 alternative drinking water facilities of clean water reservoirs (CWC) and overhead reservoirs (OHR) have been considered to evaluate the alternative drinking water facility assessment. Euclidean distance has been measured in both cases from the highly fluoride contaminated villages. Results of risk areas identification reveal that the maximum areas of Para block fall into high-risk categories whereas some portions of the south-eastern corner of Barabazar, western portions of Manbazar I, small areas of Kashipur, Purulia I, Purulia II, and Jhalda II block fall into high-risk categories. However, most of the regions of this section of the Manbhum-Singbhum Plateau have moderate to low risk in terms of fluorosis or fluoride hazards. In addition, moderate to low-risk areas cover 1584.24 km^2 ^whereas high to very high areas are 4543.09 km^2^. Among them, 414.29 km^2^ comes under high risk prone for fluorosis (Fig. 5).

**Fig. 6:**
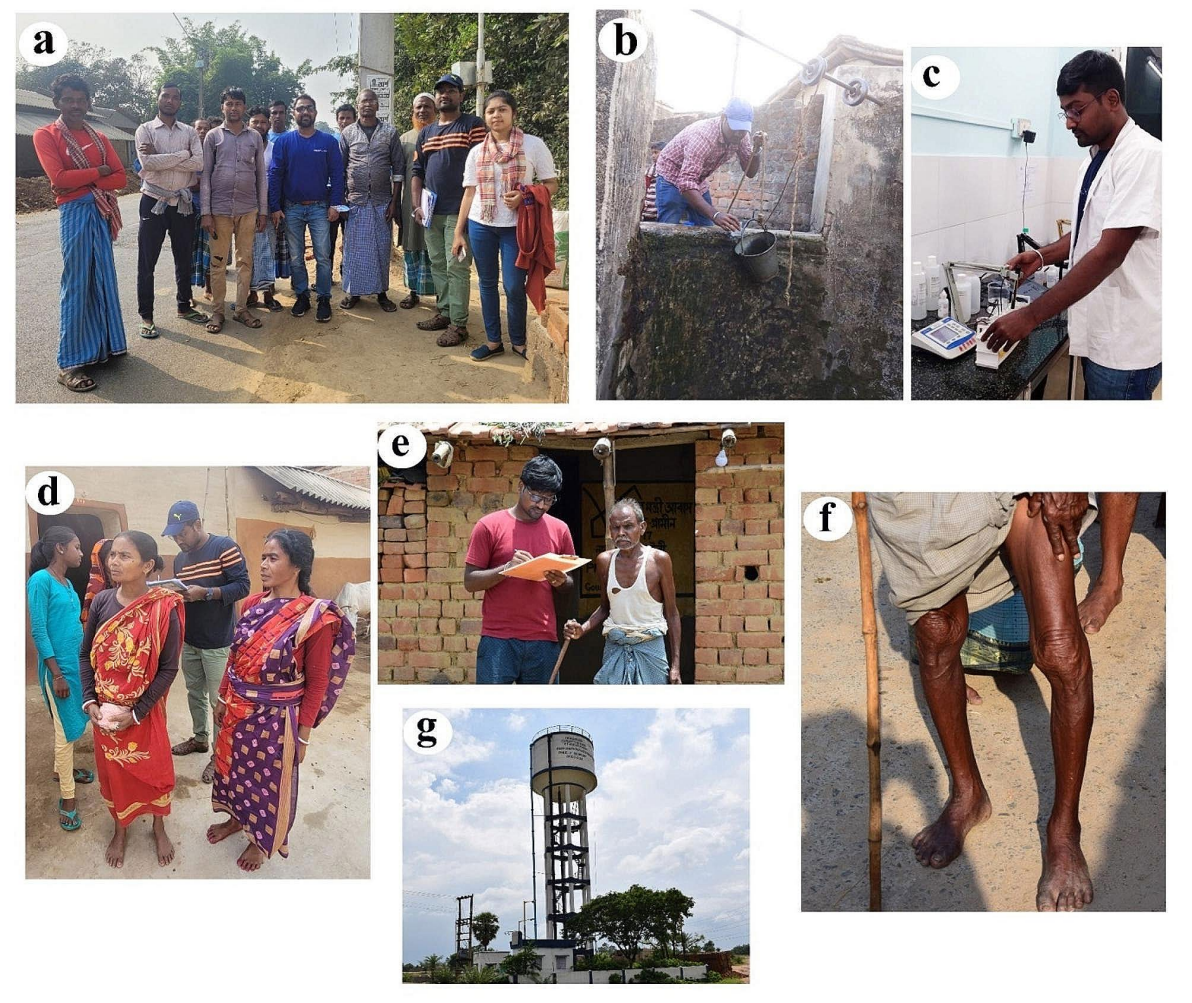
**a**) Focus Group Discussion (FGD) among the villagers **b**) Water sample collection from the drug well to assess the fluoride concentration in groundwater **c**) Sample testing in the laboratory **d**) Field investigation and survey among the dental fluorosis affected people in highest contaminated Hijla village of Barabazar **d**) Interaction with a moderate skeletal fluorosis affected aged people **f**) Moderate skeletal fluorosis affected people **g**) Alternative drinking water source, which location has been used to delineate the risk-prone zones in the study

## Discussion

**Fig. 7:**
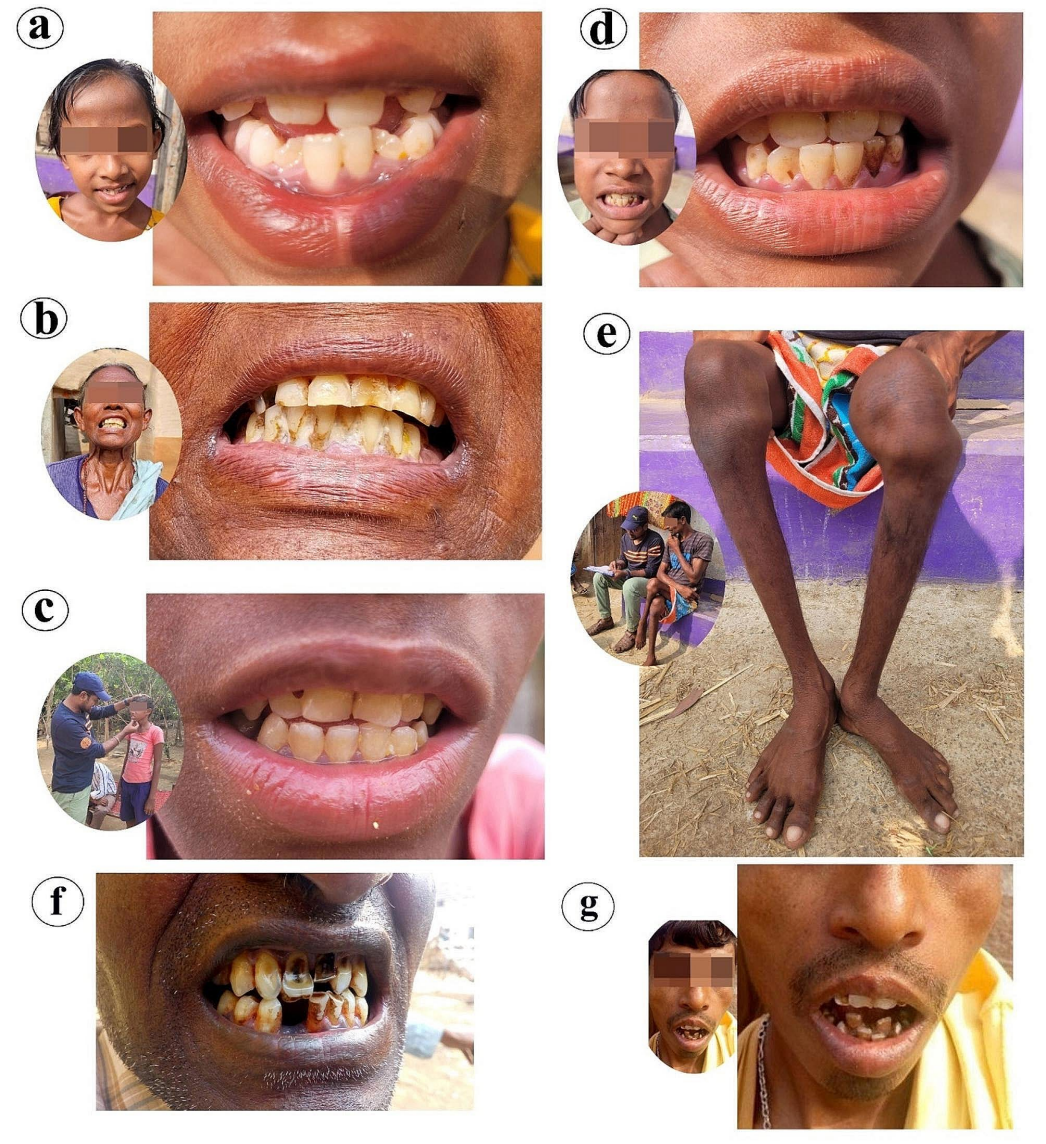
**a**) Mild dental fluorosis **b**) Severe dental fluorosis **c**) Mild dental fluorosis **d**) Moderate Dental fluorosis affected people and **e**) Moderate to severe skeletal fluorosis affected people **f**) and **g**) severe dental fluorosis people in different blocks of Purulia district

Several analytical investigations demonstrated that fluoride concentration in drinking water sources above 1.5 mg/l affects health and society [11, 13, 46, 47]. The present systematic study also strongly endorses this fact and points out that fluoride contaminated water creates numerous health problems as well as societal difficulties among the affected people in a semi-arid plateau fringe area of the Indian Subcontinent (Figs. 6 and 7). During the field survey, we measured the severity and magnitude of dental and skeletal fluorosis in high fluoride exposure areas or endemic susceptible villages. The Community Fluorosis Index indicates that there is a severe health concern in the community because the value crosses 0.6 in many villages. If values cross 0.6, then there is a public health concern in terms of fluorosis endemic [38]. Prevalence and severity are major concerns in some blocks, particularly in Barabazar, Purulia II, Raghunathpur, and Nituria blocks (Fig. 6). These areas are facing moderate to severe fluorosis endemic due to the presence of fluoride in their drinking water sources beyond the permissible limit (greater than 1.5 mg/L). Excessive intake of fluoride-rich water affects the teeth and eventually generates dental fluorosis [48] but previous studies related to the impact assessment of dental fluorosis highlighted that the possibility of non-discrete symmetrical defects of enamel could arise for other reasons [49]. However, people in high-contaminated villages face fluorosis because people knowingly or unknowingly depend on groundwater for drinking and domestic usage. People are consuming the polluted water because there is no alternative way to survive particularly in the summer season. After all, the entire region faces a severe water crisis, high fluctuations, and deterioration of groundwater storage [6]. Fluorosis occurs in semi-arid environments due to the excess intake of fluoride-contaminated drinking water for extreme weather events [50]. This study similarly designates that not only dental but also skeletal fluorosis is the foremost health concern in this semi-arid part of the Manbhum-Singhbhum Plateau. About 87 people are affected by skeletal fluorosis and suffer from stiffness in the back spine, abnormalities in ligaments and itchy sensations in limbs. Additionally, 30% of affected individuals are facing pain in the neck, shoulder and hip joints which have been measured as mild to moderate types of skeletal fluorosis. The coefficient value (0.69) between fluoride levels in villages and physical disability reveals a positive association. Therefore, fluoride in groundwater triggers the movement incapability of people in surveyed villages. They cannot have better treatment in private nursing homes because of their poor economic condition. It has been observed that the average income of surveyed villagers is not more than 3000 rupees per month (including other financial assistance from the government).

Societal consequences like school dropout, social isolation and remarriage are major concerns in these extended sections of the Manbhum-Singbhum Plateau region. This field-intensive systematic study reveals that about 149 people are facing problems in social interactions in their everyday life as they are severely affected by dental or skeletal fluorosis. Sociologists often call this as withdrawal syndrome or ‘retreatism’ which is a direct consequence of their ‘sick role’ [51]. Affected people tend to isolate themselves from their neighbors [52]that further marginalizes them from the routinized activities of village life. This is an indication of the intensity of stigmatization due to fluoride-induced deformities in these tribal-dominated areas. Even a good number of fluorosis-affected people are not encouraged to join any wedding or social festivals. However, the correlation matrix shows that intensifications in the fluoride level positively influence the social isolation in the study areas. Nagra and Jibanpur villages are substantial examples of socially isolated people affected by moderate to severe fluorosis endemic.

School dropout is also a vital issue in this region, and huge numbers of affected students have discontinued their school education at the matriculation level because of the perceived stigma that affects their relationships with others in the school. Deformities affect their self-dignity and self-actualization and create a fear of non-acceptance by others in the group. Physical appearances of people in social gatherings, which according to Goffman (1956) are important for ‘impression management ‘in a ‘front stage’, are restricted due to the fear associated with their tooth color, mostly during smiling [53, 54]. Fluoride level and school dropout have a strong positive correlation, which signify that high fluoride contamination in drinking water sources accelerates the rate of school dropouts. Consequently, students are barely interested in showing their teeth in front of others because they are ashamed of the dullness and abnormal appearance of their teeth. Similarly, they also try to avoid social gatherings and refuse to go to school. Many previous studies illustrated that aesthetic problems among young age people have been seen particularly during smiling [55, 56]. In addition, this detailed study also reveals that when parents or family members suffer from chronic skeletal and non-skeletal health disorders, older family members do not send their children to school.

Naidu et al. (2013) reveal that there is a lack of awareness among the people of low socio-economic strata [57]. Most of the tribal people do want to believe in fluoride-related health disorders and depend on indigenous methods and homeopathic treatment. Rathi (2014) highlighted that in the case of fluorosis, no ayurvedic treatment is available to cure the chronic form of illness [58]. In several cases, fluorosis-affected women are ill-treated for their crippling dental fluorosis and physical disability because of skeletal fluorosis and often these lead to divorce [13]. Some previous studies also established that disabled women have limited access to education and they are often marginalized and invisible [59, 60]. This study highlights that there is a low remarriage rate (4%) due to severe fluorosis-related deformities. However, after the assessment of the overall risk and severity of fluorosis, some blocks including Para, Barabazar, Manbazar I, Kashipur, Purulia I and II and Jhalda II are considered as high-risk-prone areas. The study also reveals that most of the affected people never go to a medical professional for treatment at the initial stage. This is indicative of lack of consciousness in this part of the Manbhum-Singhbhum Plateau. This tribal dominated demography of Jungle Mahal Blocks of Purulia district is experiencing severe fluorosis related health hazards and societal problems because of multiple reasons such as dependence on nature, lack of employment, severe economic hardship, lack of social and environmental awareness, lack of education, inadequate medical facilities, scarcity of pure drinking water, etc. This research exposed that many young people have completely deviated from the so-called ‘mainstream’ society and have been showing inclinations towards some extremists or unlawful associations due to social isolation, disability, poverty, political pressure etc. For example, Gopal Mahato (48 years old) of Hijla village, Balarampur block of Purulia, has been suffering from severe skeletal fluorosis since 2003. He is not capable of and has to depend on other family members to move from one place to another. His family depends on daily agricultural labour but now he is jobless. In the case of dental fluorosis, Anjali Mahato (9 years old,) and Radhanath Mahato (44 years old,) have been suffering dental fluorosis with brown speckles on their teeth.

## Conclusion

This study gives an outline of the detrimental effects of high fluoride levels that affect human health and society in the part of Manbhum-Singhbhum Plateau region. People living in highly contaminated villages are vulnerable to dental and skeletal fluorosis. In most of the villages in this semi-arid fluoride endemic hotspot region, the Community Fluorosis Index is greater than the prescribed value (0.6) as recognized in the Dean’s Index. Some blocks in this tribal-dominated hard rock terrain come within the very risk-prone zone category and suffer from fluorosis-induced hazards. School dropout, social isolation and remarriage are some of the remarkable social consequences which are directly related to the problem of fluoride in this terrain region. However, the present study suggests that awareness through improved education is much needed to fight against the negative impacts of fluoride on the human body and society. Some alternative sources of drinking water are now available in some localities but a wider and large-scale pipe water scheme should be in place to supply water regularly to the high fluoride-contaminated villages. It is also essential to initiate water crisis management in this semi-arid region. This situationis common in Asian countries, particularly in China and India. Therefore, this study will be helpful for better management not only in this part of the Indian subcontinent but also around the different parts of the semi-arid fluoride-contaminated regions of the world.

### Electronic supplementary material

Below is the link to the electronic supplementary material.

Supplementary Material 1

## Data Availability

Data and materials are available on request.
