# Supplementary material for: Severe magnitude of dental and skeletal fluorosis and its impact on society and environment in a part of Manbhum-Singhbhum Plateau, India
Source: BMC Public Health. 2024 Jul 23;24:1971. doi: 10.1186/s12889-024-19446-1 (PMC11267771; doi:10.1186/s12889-024-19446-1)
Supplement: Supplementary file 1 — Supplementary Material 1 [file 12889_2024_19446_MOESM1_ESM.docx]

**QUESTIONNAIRE**

Block Name: ………………. Mouza: …………………………. Village: …………………….

Gram Panchayet ………………………………….

**SECTION A. DEMOGRAPHIC CHARACTERISTICS**

**1. Name of the Respondent:**

**(a)** Age: …………………. **(b)** Sex: ……………………. **(c)** Caste: Gen/SC/ST/OBC……………

**2. Age Structure:**

| **Age group** | **No. of Male** | **No. of Female** |
| --- | --- | --- |
| Less than 15 |  |  |
| 15-30 |  |  |
| 30-45 |  |  |
| 45-60 |  |  |
| More than 60 |  |  |

**3. Status of Education:**

| Education Level | No. of Male | No. of Female |
| --- | --- | --- |
| Illiterate |  |  |
| Up to class IV |  |  |
| Up to class X |  |  |
| Madhyamik Pariksha |  |  |
| Higher Secondary |  |  |
| Graduate |  |  |
| Post Graduate |  |  |
| Other Qualification |  |  |
| Drop out |  |  |

**4. Economic status:**

**(a)** Family occupation: Major…………………… Other……………………….

(i) Marginal farmer ( ) (ii) cultivator ( ) (iii) agricultural labour ( ) (iv) seasonal worker ( ) (v) daily labour ( ) (vi) business ( ) (vii) service – Private/Govt. ( )

**(b)** Occupational Structure:

| Occupation | Male | | | Female | | | Remarks |
| --- | --- | --- | --- | --- | --- | --- | --- |
|  | <20 | 20-40 | >40 | <20 | 20-40 | >40 |  |
| Marginal Farmer |  |  |  |  |  |  |  |
| Cultivator |  |  |  |  |  |  |  |
| Agricultural Labour |  |  |  |  |  |  |  |
| Seasonal Worker |  |  |  |  |  |  |  |
| Daily Labour |  |  |  |  |  |  |  |
| Business |  |  |  |  |  |  |  |
| Service – Private/Govt |  |  |  |  |  |  |  |

**(c)** No of total family members and earning members: Male…………. Female………….

**(d)** Family monthly income: (i) Less than 3000 (ii) 3000 – 6000 (iii) 6000-9000 (iv) 9000-12000 (v) More than 12000

**(e)** Duration of work: (i) Less than 3 months ( ) (ii) 3 to 6 months ( ) (iii) > 6 months ( )

**(f)** Nature of land tenancy: (i) Own land ( ) (ii) Share cropper ( )

**(g)** Land holding capacity: Agricultural land ……………… Other land ……………...

**(h)** Cropping pattern and Major crops:

**(i)** Sources of irrigation: (i) River ( ) (ii) Pond ( ) (iii) Tube-Well ( ) (iv) Deep Tube-Well ( ) (v) Submersible pump ( )

**(j)** Household gadgets: TV, land line phone, Cell phone, fridge, bicycle, motor cycle, car, radio, computer with/without internet, water purifier

**SECTION B. WATER QUALITY AND MAGNITUDE OF FLUOROSIS**

**5. Sources and Quality of Water:**

**(a)** Sources of drinking water: (i) Tube-well ( ) (ii) Submersible pump ( ) (iii) Public tap ( ) (iv) Dug well ( ) (v) River/Dam ( ) (vi) Pond ( )

**(b)** Location of tubewell/dug well: ……………………………………………………………….

**(c)** No. of fluoride contaminated tubewell/dug well in your locality:

**(d)** Depth from which ground water is extracted……………………………………………

**(e)** Quality of drinking water: (i) Good ( ) (ii) Bad/Polluted ( ) If it is polluted, mention the source of pollution according to respondents view.……………………………………………….

**(f)** Whether water is fluoride affected? Yes/No

……………………………………………………………………………………………………..

**(g)** Anthropogenic causes of fluoride in the locality: (i) Burning of coal ( ) (ii) manufacturing processes of aluminum ( ) (iii) manufacturing processes of steel ( ) (iv) manufacturing processes of bricks ( ) (v) phosphatic fertilizers industries ( )

**6. Types and Magnitude of Fluorosis**:

**(a)** Types of fluorosis (i) Dental: ( ) (ii) Skeletal: ( )

**(b)** Age-sex structure of fluorosis affected people -

| Types of Fluorosis | Male | | | | Female | | | |
| --- | --- | --- | --- | --- | --- | --- | --- | --- |
|  | <10 | Oct-30 | 30-60 | >60 | <10 | Oct-30 | 30-60 | >60 |
| Dental |  |  |  |  |  |  |  |  |
| Skeletal |  |  |  |  |  |  |  |  |

**(c)** Symptoms: (i) Aches and pain in the joints ( ) (ii) non-ulcer dyspepsia ( ) (iii) Polyurea and polydipsia ( ) (iv) muscle weakness ( ) (v) fatigue ( ) (vi) anemia ( ) (vii) very low hemoglobin ( ) (viii) mild dental fluorosis ( ) (ix) moderate dental fluorosis ( ) (x) severe dental fluorosis ( )

**(d)** Long term effects of fluoride contamination/fluorosis: ………………………………………..

**(e)** Number of members suffering from fluorosis in the family: ……………………………………

**(f)** Number of people affected by fluorosis in the village: ………………………………………….

**SECTION C. FOOD HABIT, MEDICAL FACILITY AND MANAGEMENT**

**7. Food habit**

**(a) Frequency of food intake:** (i) one time ( ) (ii) two time ( ) (iii) three time ( )

(iv) four time ( ) (v) nil ( )

**(b) Types of food:**

(i) Protein rich food ( ) (ii) Mineral rich food ( ) (iii) Vitamin rich food ( ) (iv) Others ( )

**(c) Amount of fluoride rich water they drink daily:**

**8. Medical Assistance:**

**(a) Types:** (i) PHC ( ) (ii) Govt. Hospital ( ) (iii) Private clinic ( ) (iv) Others ( ) ………………………………………………………………………………………………………

**(b) Availability of doctors:** (i) One ( ) (ii) two ( ) (iii) three ( ) (iv) none ( )

**(c) Types of doctor:** (i) Dental surgeon ( ) (ii) Orthopedic ( ) (iii) others ( )

**(d) Types of doctor consulted:** (i) Allopathic ( ) (ii) Homeopathic ( ) (iii) Ayurvedic ( ) (iv) Quack ( )

**(e) Distance of Govt. hospital/primary health centre from their locality:**

**(f) Mode of transport to reach Govt. hospital/Public health centre:** (i) Bus ( ) (ii) Car ( ) (iii) Bike ( ) (iv) Bi-cycle ( ) (v) By walking ( ). ……………………………

**9.** Any preventive measures taken by local administrative body: ………………………………….

………………………………………………………………………………………………………

**10.** Is there any water harvesting measures implemented in your locality? If yes, then what types of measures has been implemented? ………………………………………………………………

**11.** Is there any water supply from Dam and reservoirs in the locality? If yes, then write down the name of the Dam and quality of Dam water they receive:

………………………………………………………………………………………………………

**12.** Is there any underground dam situated in the village/Municipality? Yes ( ) No ( )

**13.** Is there any arrangement of surface water storage/ artificial recharge in the locality? If yes, then write down the type of arrangement?

…………………………………………………………………………………………………………………………

**14.** Is there any campaigning from Govt. to grow awareness of local people? Yes ( ) No ( )

**15.** Is there any health check-up programme? (i) Yes ( ) (ii) No ( ) If yes, then mention the nature of health check-up programme: (i) Monthly ( ) (ii) Quarterly ( ) (iii) Yearly ( )

…………………………………………………………………………………………………………………………..

**16.** Awareness level of local people: (i) High ( ) (ii) Moderate ( ) (iii) Low ( )

……………………………………………………………………………………………………..
